# Quantifizierung von Ki-67 in PitNET („pituitary neuroendocrine tumors“)/Adenomen

**DOI:** 10.1007/s00292-024-01319-4

**Published:** 2024-07-11

**Authors:** Judith Klein, Kai Saeger, Wolfgang Saeger

**Affiliations:** 1 Institut für Neuropathologie der Universität Hamburg, UKE, Martinistraße 52, 20246 Hamburg, Deutschland; 2 VMScope GmbH, Berlin, Deutschland

**Keywords:** PitNET/Hypophysenadenome, Ki-67-Index, Digitale Bildanalyse, Hypophyse, Proliferation, PitNET/adenoma, Ki-67-index, Digital image analysis, Pituitary gland, Proliferation

## Abstract

Die vorliegende Studie vergleicht erstmals die Bestimmung des Ki-67-Index bei PitNET/Hypophysenadenomen durch Pathologen mit einer computergestützten Methode (Cognition MasterSuite, Fa. VMScope, Berlin). PitNET/Hypophysenadenomen weisen häufig einen geringen Proliferationsindex aus. Eine hohe Observervariabilität besteht v. a. bei der Schätzung in diesem niedrigen Prozentbereich. Eine zuverlässigere Festlegung wäre durch das 4‑Augen-Prinzip möglich, das jedoch nicht kontinuierlich zu realisieren ist. Abhilfe verspricht hier die digitale Bildanalyse. In der Untersuchung konnte eine deutliche Übereinstimmung der Ki-67-Schätzung durch zwei erfahrene Pathologen und der Bestimmung mit Hilfe der digitalen Bildanalyse gezeigt werden. Das digitale Bildanalysesystem ist hervorragend zur Bestimmung der Proliferationsrate von PitNET/Hypophysenadenomen geeignet und kann daher das „dritte“ und „vierte Auge“ darstellen.

## Abstract (en)

This study is the first to compare the determination of the Ki-67 index in pituitary neuroendocrine tumors (PitNET)/pituitary adenomas by pathologists with a computerized method (Cognition MasterSuite from VMScope, Berlin, Germany). PitNET/pituitary adenomas often show a low proliferation index. Observer variability is high, especially when estimating in this low percentage range. A more reliable determination would be possible using the four-eyes principle, but this cannot be realized continuously; thus, digital image analysis is a promising solution. In the study, there was clear agreement between the Ki-67 estimate by two experienced pathologists and the determination with the aid of digital image analysis. The digital image analysis system is excellent for determining the proliferation rate of PitNET/pituitary adenomas and can therefore be used to determine the “third” and “fourth eye”.

## Hintergrund und Fragestellung

Bei Obduktionen können in etwa 10 % der Hypophysen Mikroadenome nachgewiesen werden, die klinisch nicht ersichtlich waren und in der Regel keine biologische Relevanz besitzen [2]. Symptomatische Hypophysentumoren sind im Vergleich dazu sehr viel seltener [12]. In der Regel werden diese in Deutschland chirurgisch behandelt; jährlich werden etwa 1500 Eingriffe durchgeführt [7]. Eine Ausnahme stellen die häufigen Prolaktinome dar, welche gut auf Dopaminantagonisten ansprechen [17].

Die PitNET/Adenome (Pituitary Neuroendocrine Tumors/Adenome) stammen aus neuroendokrin differenzierten Zellen des Hypophysenvorderlappens ab und werden nach der aktuellen WHO-Klassifikation von 2022 anhand ihrer Hormonexpression und molekularer Marker klassifiziert. Es ist jedoch trotz intensiver Forschung bis zum jetzigen Zeitpunkt nicht gelungen, klare feingewebliche Kriterien, die eine Aussage zum biologischen Verhalten der PitNET/Adenomen erlauben, zu definieren. Angaben zur Rezidivneigung, oder dazu, ob eine zu einer Metastasierung neigende Erkrankung vorliegt, können am Operationspräparat nicht getroffen werden [1].

In der wissenschaftlichen Diskussion um die Identifikation prognostischer Marker im histologischen Untersuchungsmaterial wurde in der Vergangenheit intensiv über Bedeutung der immunhistologischen Reaktion für Ki-67 und p53 diskutiert [13, 20]. Diese Kontroverse spiegelt sich auch in den WHO-Klassifikationen der Hypophysenneoplasien wider. In der 3. und 4. Aufl. der WHO-Klassifikation wurden noch ein Ki-67-Wert von mindestens 3 % und eine starke p53-Expression als Hinweise auf einen potenziell malignen Tumor (damals als sog. atypisches Hypophysenadenom) gewertet [4, 14]. In der aktuellen Ausgabe der WHO-Klassifikation wird lediglich darauf hingewiesen, dass Ki-67 regelmäßig im Rahmen der Diagnostik von PitNET/Adenomen bestimmt und im Befundbericht angegeben werden soll [1].

Häufig weisen PitNET/Adenome nur sehr geringe Ki-67-Werte aus [6], die Abschätzung des prozentualen Werts im niedrigen einstelligen Prozentbereich ist erfahrungsgemäß schwierig und birgt die Gefahr einer kritischen intra- und interindividuellen Observervariabilität. Eine zuverlässigere Festlegung wäre durch das 4‑Augen-Prinzip möglich, das jedoch aufgrund des Personalmangels und der hohen Arbeitsbelastung nicht kontinuierlich zu realisieren ist. Abhilfe verspricht hier die digitale Bildanalyse.

Um zu prüfen, ob ein digitales Bildanalysesystem eine zuverlässigere Schätzung von Ki-67 bei PitNET/Adenomen ermöglicht, führten wir unsere Studie durch.

## Studiendesign und Untersuchungsmethoden

Das Untersuchungsmaterial entstammt dem Hypophysentumorregister der Deutschen Gesellschaft für Endokrinologie (DGE), angesiedelt am Institut für Neuropathologie des Universitätsklinikum Hamburg-Eppendorf (UKE), und umfasst sowohl Resektate von PitNET/Adenomen der Klinik für Neurochirurgie des UKE sowie auch zur Referenz übersandte Proben. Insgesamt wurden 206 Fälle aus den Jahren 2013 bis 2015 eingeschlossen. Die Schnittpräparate wurden wie die konventionelle Färbung und auch die immunhistologischen Untersuchungen ausschließlich am Institut für Neuropathologie des UKE angefertigt.

Im ersten Schritt erfolgte die Schätzung des Ki-67-Index aller 206 Fälle durch 2 voneinander unabhängige Pathologen.

**Abb. 1:**
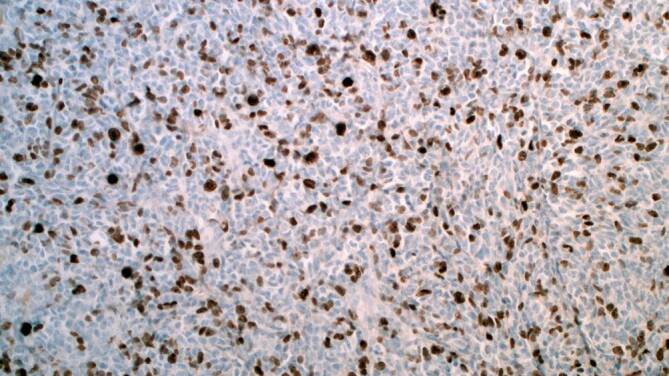
Immunhistologische Reaktion Ki-67 (40 ×)

Anschließend wurden zur Vorbereitung der digitalen Analyse von jedem Fall jeweils 3 repräsentative digitale Fotografien aus unterschiedlichen Abschnitten der immunhistologischen Präparate für Ki-67 erstellt (Abb. 1).

Von 10 zufällig ausgewählten Fällen wurden aus dem digitalen Bild zunächst alle Tumorzellen und anschließend alle in der Ki-67-Reaktion positiven Zellen manuell ausgezählt.

Für die darauf folgende digitale Bildanalyse wurde der Ki-67-Quantifier der Cognition Professional Master Suite (Fa. VMscope, Berlin, Deutschland) an allen 206 Fällen angewendet. Das System wurde im Jahr 2015 im Rahmen der „GeparTrio Breast Cancer Study“ zur Ki-67-Auswertung an Stanzbiopsien von Mammakarzinomen evaluiert [8].

Bei der praktischen Anwendung wird kein vorheriges Training, eine spezielle Kalibrierung oder eine sonstige Interaktion mit dem Anwender vorausgesetzt. Die Auswertung kann an digitalen Fotografien mikroskopischer Bilder oder auch gescannten Schnittpräparaten erfolgen. In der vorliegenden Studie wurden digitale Fotografien verwendet, da die technisch neuere Methode des Scannens von Schnittpräparaten noch nicht in der Breite etabliert ist.

**Abb. 2:**
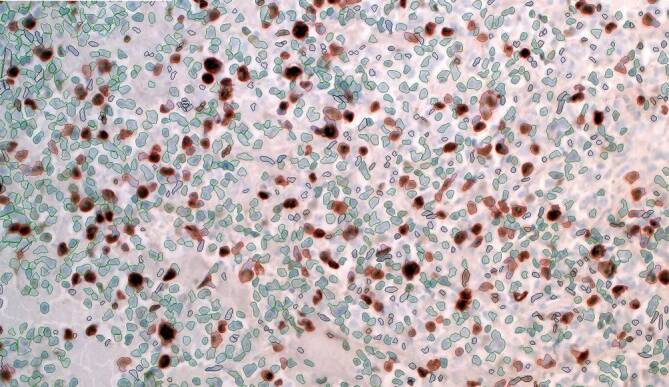
Auswertung der Reaktion Ki-67 mit dem Quantifier. *rot* positive Tumorzellen, *grün* negative Tumorzellen

Die Ergebnisausgabe erfolgt innerhalb weniger Sekunden. Neben den absoluten und prozentualen Werten der Gesamttumorzellzahl sowie positiv gefärbter Tumorzellen gibt das digitale System ein Bild mit der visuellen Darstellung aller ermittelten Zellen aus. Hierbei werden Zellen ohne Reaktion für Ki-67 grün und solche mit positiver Reaktion rot dargestellt (Abb. 2).

Aus technischen Gründen waren nicht alle Fotografien einwandfrei digital auswertbar. Es wurden daher nur solche Fälle mit mindestens 2 digital auswertbaren Bilden berücksichtigt. In die statistische Auswertung wurden 148 der insgesamt 206 Fälle einbezogen.

Zur statistischen Analyse wurde der Intraklassenkorrelationskoeffizient nach Pearson, der den Grad der Übereinstimmung der ermittelten Werte angibt, verwendet [9]. Die graphische Darstellung der Abweichungen der Messwerte vom jeweiligen Differenzmittelwert erfolgte mit Hilfe von Bland-Altman-Plots. Außerdem wurden die Ergebnisse in Kreuztabellen dargestellt und hieraus wurde die Accuracy („Genauigkeit der Messung“) bestimmt.

## Ergebnisse

Der Intraklassenkorrelationskoeffizient nach Pearson (ICC) zeigt eine hohe Übereinstimmung der Ki-67-Bestimmung. Beim Vergleich der Schätzung durch 2 Pathologen (sog. „eyeballing“) und der Bestimmung durch das digitale System beträgt er 0,967. Wird das digitale Analysesystem mit Ki-67-Werten, die durch genaues Auszählen der Gesamtzellzahl und der positiven Zellen ermittelt wurden (10 repräsentative Fäll), abgeglichen, ergibt sich ein ICC von 0,938.

**Abb. 3:**
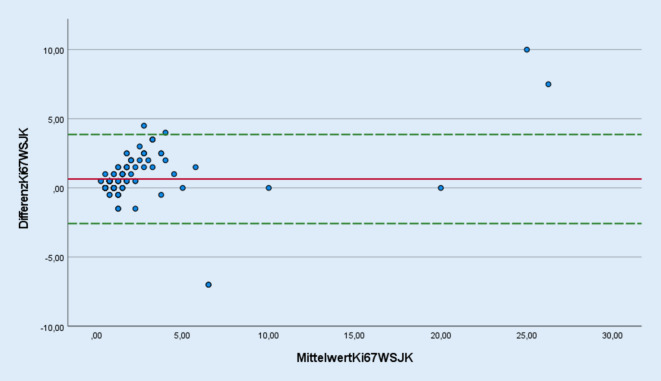
Bland-Altman-Plot für die Ki-67-Schätzung durch beide Pathologen (JK und WS). Die beiden *gestrichelten, grünen Linien* entsprechen der oberen und unteren Grenze (Differenzmittelwert +/- 1,96*Standardabweichung). Die *rote Linie* entspricht dem Differenzmittelwert der Messung durch beide Untersucher

**Abb. 4:**
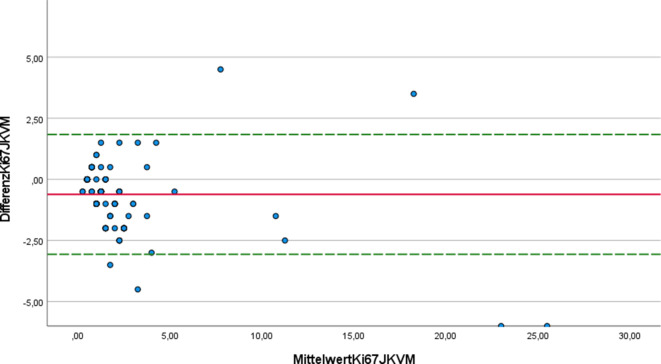
Bland-Altman-Plot für die Ki-67-Schätzung durch einen Untersucher (JK) und das digitale System (VM)

Mithilfe der Bland-Altman-Plots, die die Messungen der Pathologen (Abb. 3) als auch der einzelnen Pathologen und dem digitalen System (Abb. 4) graphisch gegenüberstellen, konnte gezeigt werden, dass die Ergebnisse der Ki-67-Schätzungen jeweils in einem tolerablen Bereich um den Mittelwert der Ergebnisdifferenz schwanken.

Mit Hilfe von Kreuztabellen können Gesamthäufigkeiten und bedingte Häufigkeiten zweier Ereignisse bzw. Schätzungen zusätzlich dargestellt werden. Zur Veranschaulichung werden im Folgenden zunächst die Ki-67-Schätzungen beider Pathologen und anschließend die digital ermittelten Werte mit denen eines menschlichen Untersuchers in tabellarischer Form verglichen. Angegeben sind in der oberen Zeile jeweils die Schätzungen durch einen menschlichen Untersucher, gegen die die Ergebnisse des zweiten Untersuchers bzw. des digitalen Systems abgetragen wurden. Im mittleren Teil der Tabelle finden sich die bedingten Häufigkeiten einer übereinstimmenden und auch einer gegensätzlichen Schätzung.

Bei der Gegenüberstellung der Ki-67-Schätzung durch die beiden Untersucher zeigt sich, dass der Untersucher WS in 124 und die Untersucherin JK in 138 der 148 Fälle die Proliferationsrate auf < 3 % schätzte (Tab. 1). Es konnte eine hohe Übereinstimmung für den Fall, dass ein Untersucher einen geringen Ki-67-Wert angab, ermittelt werden. Die Genauigkeit der Ki-67-Schätzung durch beide Untersucher beträgt 0,85.

**Tab. 1:** Kreuztabelle für die Ki-67-Schätzung durch beide Pathologen (JK und WS)

		Ki-67 JK		
		≤ 3 %	> 3 %	Gesamt
*Ki-67 WS*	*n*	122	2	124
*≤* *3* *%*	% von Ki-67 WS	98,4 %	1,6 %	100,0 %
–	*n*	16	8	24
*>* *3* *%*	% von Ki-67 WS	66,7 %	33,3 %	100,0 %
*Gesamt*	*n*	138	10	148
	% von Ki-67 WS	93,2 %	6,8 %	100,0 %

Das digitale System gab in 122 von 148 Fällen einen Ki-67-Prozentwert von < 3 % an (Tab. 2). In den Fällen, in denen die Untersucherin JK einen geringen Ki-67-Wert schätzte, gab in 88 % der Fälle das digitale Bildanalysesystem einen gleichartigen Bereich an. Bei einem Ki-67-Wert von ≥3 % gab das digitale System in 90 % der Fälle einen gleichartigen Wert wie die Untersucherin aus. Die Genauigkeit der Ki-67-Schätzung durch die Untersucherin und das digitale System beträgt 0,87.

**Tab. 2:** Kreuztabellen für die Ki-67-Schätzung durch einen Pathologen (JK) und das digitale System (VM)

		Ki-67 VM		
		≤ 3 %	> 3 %	Gesamt
*Ki-67* *JK*	*n*	121	17	138
*≤* *3* *%*	% von Ki-67 JK	87,7 %	12,3 %	100,0 %
–	*n*	1	9	24
*>* *3* *%*	% von Ki-67 JK	10,0 %	90,0 %	100,0 %
*Gesamt*	*n*	122	26	148
	% von Ki-67 JK	82,4 %	17,6 %	100,0 %

## Diskussion

Die vorliegende Untersuchung wurde zur Evaluation eines digitalen Bildanalysesystems bei Einsatz in der Bestimmung der Proliferationsrate (Ki-67-Index) an PitNET/Adenomen durchgeführt. Das Ergebnis dieser digitalen Berechnung wurde mit der quantitativen Schätzung durch 2 Pathologen abgeglichen.

Bei der Bestimmung der Proliferationsrate mit Hilfe der immunhistologischen Reaktion von Ki-67 bestehen beim Allgemeinpathologen breite Erfahrungen in der Auswertung, im Fall von Ki-67 häufig aus der Diagnostik an Mammakarzinomen. Speziell in der Auswertung von Mammastanzzylindern sind Bestimmungen eher mittlerer Proliferationsraten und deren Abgrenzung gegenüber geringen und hohen Werten wichtig [16]. Schwierigkeiten ergeben sich bei neuroendokrinen Neoplasien v. a. bei der Schätzung sehr geringer Ki-67-Indizes, wie sie häufig bei PitNET/Adenomen zu sehen sind [5].

Die 5. Auflage der WHO-Klassifikation [1] endokriner Tumoren empfiehlt die regelmäßige Bestimmung des Ki-67-Index bei der Diagnostik von PitNET/Adenomen. Definierte Schwellenwerte sind im Gegensatz zu den vorherigen Auflagen der Klassifikation nicht vorgesehen. Ursächlich hierfür ist, dass für den Marker in der Vergangenheit im Rahmen wissenschaftlicher Studien widersprüchliche Ergebnisse für dessen prognostische Bedeutung gewonnen wurden [10, 11, 15, 18].

In einer Arbeit von Chiloiro et al. [3] werden wahrscheinliche Gründe für die diskrepanten Studienergebnisse genannt. Neben einem Vergleich verschiedener Adenomtypen, unterschiedlich definierter Patientengruppen wurde von den Autoren auch die bislang unklare Definition eines aggressiven Hypophysentumors als kritische Punkte gesehen. Als ein weiterer Grund für die unterschiedlichen Studienergebnisse wird die Stichprobengröße angeführt. Letztgenannte Beobachtung wird auch durch die Ergebnisse einer Untersuchung von Volynskaya et al. aus dem Jahr 2019 unterstrichen [19]. Es konnte hier an neuroendokrinen Neoplasien gezeigt werden, dass bei einer Erhöhung der Zahl ausgewerteter Zellen der Ki-67-Index eher in Richtung niedrigerer Werte tendiert. Dies trifft nach Angaben der Autoren speziell auf Proliferationsindizes in unteren Bereichen zu. Vor diesem Hintergrund wirkt sich die Probenart auf die Höhe der Proliferationsfraktion aus: Die Auswertung vieler kleiner Biopsien erhöht den Ki-67-Wert im Vergleich zu einem Resektat. Schließlich wird von Chiloiro et al. [3] auch der Modus der Beurteilung als Grund für divergente Studienergebnisse angeführt. Neben den bekannten Unterschieden zwischen der „Hot-spot“-Methode und der Betrachtung des Gesamtpräparats spiele es eine Rolle, ob konventionell oder digital ausgewertet werde. Dem letztgenannten Punkt widmet sich die vorliegende Arbeit.

## Fazit für die Praxis

- Durch die Untersuchung konnte eine hohe Übereinstimmung zwischen der digitalen Bildanalyse und der manuellen Schätzung von Ki-67-Werten bei PitNET/Adenomen der Hypophyse gezeigt werden. Der Einsatz des Quantifiers kann daher eine sehr sinnvolle Ergänzung in der täglichen Diagnostik sein.
- Das 4‑Augen-Prinzip erfahrener Pathologen ist aufgrund der Schwierigkeiten der Ki-67-Bestimmung eigentlich zu fordern, aber in Anbetracht der personellen Situation nicht durchführbar.
- Der Quantifier könnte vor diesem Hintergrund das „dritte“ und „vierte Auge“ dieses Prinzips darstellen.
